# Evaluation of the level of biomolecules isolated from date palm seeds (*Phoenix dactylifera*) and *in vitro* Antioxidant property

**DOI:** 10.37796/2211-8039.1017

**Published:** 2020-06-05

**Authors:** Najla Bentrad, Rabéa Gaceb-Terrak

**Affiliations:** Laboratory Research on Arid Zones, Faculty of Biological Sciences, Department of Biology and Physiology of Organisms, University of Sciences and Technology Houari Boumediene (USTHB), BP 32, 16111 El-Alia, Bab Ezzouar, Algiers Algeria

**Keywords:** Date palm seeds, polyphenols, TLC, HPLC-DAD, antioxidant activity

## Abstract

Date palm fruits and by-products such as seeds are a source of various elements with significant nutritional values like fibres, minerals, essential fatty acids, amino acids and phenolic compounds. The experimental part was carried out on date palm seeds from Bent Kbala cultivar, the chemical composition of the organic fraction was determined using the method of UV-visible spectrophotometer, thin layer chromatography (TLC) and high-performance liquid chromatography-diode array detection (HPLC-DAD).

The results revealed the presence of catechic tannins and approximately 17 phenolic compounds, including two compounds, which were identified for the first time in the date palm sub-product, especially in seeds such as naringenin and rutine. The assessment of the antioxidant potential shows that date palm seeds have a significant potential compared to standard antioxidants commonly used in cosmetics and neutraceutics industries.

Plants have been extensively investigated for its antioxidant properties, since phenolic compounds are widely used and considered to be potential sources of antioxidants [1]. The interest in natural antioxidants, especially polyphenolic compounds, continues to increase, these secondary metabolites are consumed daily by dietary intakes. There are many industrial requirements for use of natural sources of antioxidants:

High concentration of active molecules, adequate food supply, regular and, if possible, non- seasonal, good raw material conservation, reasonable cost, easy use, process of extraction, low aromatic character of the preparations, absence of toxicity in the extract, authorization and legal use in the food, pharmaceutical and cosmetic industries. In the process of oxidative stress regulation, flavonoids can act in various ways, it has been shown that flavonoids act *in vitro* to reduce dehydroascorbic acid via glutathione, against which they act as donors of hydrogen [2–3].

The selection of a convenient antioxidant is based on the properties required such as efficiency, solubility, heat stability and the nature of the food to be protected. In general, their antioxidant efficacy is due to their high flavonol and flavanol content, in particular epigallocatechin [4]. The search for new sources of natural antioxidants from by-products is also aimed at agricultural and food industries [5] for example cereal bran [6], fruit pips and citrus fruits [7] and grape berries in particular [8].

The beneficial nutritional values of the date palm fruit (*Phoenix dactylifera*) have long been claimed for consumption and human health [9]. Date palm seeds were considered as date fruit waste and it is now used as animal feed [10]. On the other side, date fruit seeds are used as food supplements [11] and have been roasted and milled and taken as coffee drink. The seed coffee powder meal has actually been widely marketed [11–12]. In traditional medecines, powdered seeds, is used as eye-shadow [11].

Also, it has been shown that the natural date seeds extract has a strong ability to inhibit *Pseudomonas* ATCC 14209 B1 phage infectivity (infectivity), which is known to be resistant to disinfection [13]. According to another research, extracts isolated from date palm sub-products have shown significant anti-mictrobial potential [14–16].

The aim of the present study is firstly, to identified phenolic compounds from date palm seeds (by-products) using Spectrophotometer UV-Visible, TLC and HPLC-DAD. Secondly, to determine *in vitro* antioxidant properties by evaluation of the anti-radical activity of organic extracts of seeds and antioxidants standards by the method using 2,2-diphenyl-1-picryl- hydrazyl (DPPH) considered relatively as a stable free radical.

## Materials & methods

### Plant material

Date palm fruit of Bent Kbala (BK) cultivars were collected from the region of Metlili, Ghardaia in Algeria (32° 16″ North, 32° 16″ East). The seeds were obtained by removing the endocarp, while the teguments around the seeds were preserved. The seeds (BK) were reduced to very fine powder by an electric grinder (type KSW 445 CB) and stored in a hermetically sealed bottle, protected from moisture and light.

### Total polyphenols

A maceration of 5 g of fine seeds powder in 100 mL pure methanol (99% MeOH) was performed for 24 hours under constant stirring on an agitator type “Heidolph Promax 2020″ at 140 stirring min^−1^. The obtained methanol extracts are filtered using a Buchner funnel and stored for 48 hours in the refrigerator at 6 °C. The quantitative determination of total polyphenols was carried out in accordance with the colorimetric method using Folin- Ciocalteu reagent after the addition of sodium bicarbonate at 20% [17].

The absorption of phenolic acid standards and seeds extract at 765 nm was read using the spectrophotometer “JENWAY 7305 UV/VIS”. The con-centration of phenolic compounds and tannins is determined by the equation of reference molecules such as gallic acid and catechin tannin calibration curve. The results are expressed in μg gallic acid equivalent per gram of dry wight (GAE.g^−1^ DW) for phenolic acids and catechin equivalents (CE.g−^1^ DW) for condensed tanins. Antioxidant tests were also used in this fraction.

### Catechin tannins

Under these conditions, tannins are condensed into red precipitation, which confirms their presence in the medium. In order to distinguish between the two types of tannins (Gallic or catechic tannins), we used the Stiasny reagent, which is based on the Diallo method [18]:

For 10 mL of extract, we added 5 mL of the Stiasny reagent, followed by 15 minutes of heating in a water bath at 90 °C. A revelation of greenish or blackish blue coloration of the extract results from the presence catechin or gallic tannins.

### Extraction of flavonic aglycones

The seeds extract is obtained according the protocol optimized by Lebreton and co-workers [19]:

Approximately 1g of dry vegetable powder is hydrolyzed with 80 mL of hydrochloric acid (2N), the mixture was heated in a bath of boiling water for 40 min. After cooling, the hydrolyzed powder is filtered and then transferred to a separating glass funnel, the separation is carried out twice (20-20 mL) with diethyl ether.

Following the separation of organic phase containing active compounds, it is evaporated under a ventilated host and then taken into pure methanol. This fraction is used for phytochemical investigation to recognize bioactive compounds by Thin Layer Chromatography (TLC) and High Performance Liquid Chromatography (HPLC-DAD). In addition, antioxidant tests were carried out using the bioactive compounds of this fraction (flavonic aglycons). In our previous work, the determination of the chemical content of aglycones was already carried out using Gas chromatography–mass spectrometry [15–16].

### Thin layer chromatography (TLC) analysis

TLC is a semi-qualitative analytical method that allows chemical compounds to be separated and partially identified in a complex mixture. The separation of the flavonoids was carried out by a single-dimensional TLC on silica gel F_254_ with a thickness of 0.2 mm and an aluminum support of 20 × 20 cm, which was developed in a mobile phase using the solvent system (eluent): Acetic acid/Chloroform (9-1: v/v).

Following separation, the partial identification of the flavonoids is carried out by comparing the calculated frontal ratio (FR) of each substance to the standards or molecules [20] under the ultraviolet light of the UV lamp at two wavelengths of 365 and 254 nm.

The disclosure was performed with ammonia vapor (NH_3_), the color reaction allows an unknown substance to be partially connected to a class of phenolic compounds. The flavonoids fluorescence that gives us information about the various phenolic classes. Each substance is characterized by FR that corresponds to the ratio between the molecule displacement (d) and the solvent front displacement (D).

### High performance liquid chromatography (HPLC-DAD) analysis

The chromatograph is equipped with a brand system the Agilent 1100 series, a quaternary pump and an automatic injector. The column is of the type Hypersil™ BDS C_18_, 5 μm, 250 4,6 mm at 30 °C. The mobile phase consists of water acidified with acetic acid (0.2%) at a pH of 3.1 and acetonitrile with a linear elution gradient for 30 minutes at 1 mL/min. The detection of separable substances is based on a diode array detector (DAD Diode Array Detector), which enables both to measure absorbance directly on a plurality of wavelengths: 200, 254, 280, 330 and 355 nm selected according to the molecules maximum absorbance.

### Evaluation of the antioxidant activity

The study of the anti-radical activity of seeds extracts from the date palm and natural antioxidants of reference was carried out in accordance with the method described by Blois [21] using 2,2-diphenyl-1-picryl-hydrazyl (DPPH), which it is a relatively stable free radical. Antioxidants reduce the violet color of DPPH to a yellow color, diphenyl-picrylhydrazine, whose color intensity is inversely proportional to the antioxidant's ability to give protons in the mixture. The DPPH radical is an oxidant that the antioxidant potential can be measured at 517 nm.

A 0.3 mM solution of DPPH is prepared in methanol according to the protocol established by Gulcin et al. [22] and adapted by Bouhlali et al. [23]; 800 μL of this solution are added to 2 mL of reference antioxidant. Our choice has been made with known natural antioxidants: Vitamin E (10 μg mL^−1^), ferulic, gallic and L-ascorbic acids (5 μg mL^−1^). The organic seeds extract was assessed for its antioxidant potential. As before, 800 μL of DPPH solution (0.3 mM) is added to 2 mL of seeds extract. The following formula is used to estimate the anti-radical potential of crude extract from plant or reference substances as follows:

$$\begin{array}{l} (I\%)=\left\{\left(A_{0}-A_{1}\right)/A_{0})\times 100\right\}\\ \;\text{I\%: Percent inhibition of DPPH}\\ \;A_{0}:\text{Absorbance of white}\;(\text{methanol})\\ \;A_{1}:\text{Absorbance of the tested sample}\;\left(\text{organic extract or reference molecule}\right) \end{array}$$

### 50% inhibition concentration: IC_50_

After spectrophotometric determination at a wavelength equal to 517 nm, the concentration that trapped 50% of the radical DPPH or the 50% inhibition concentration designated IC_50_ is graphically determined.

### Statistical analysis

The experimental data are expressed in standard error mean (m ± SEM) from n separate experiments and the significant differences between the experiments were recorded at p < 0.05.

## Results & discussion

### Detection and identification of phenolic compounds Quantification of phenolic compounds and catechin tanins

According to this work, the seeds containing a low amount of phenolic acids, it is approximately estimated at 2.5 ± 0.453 mg.g^−1^ gallic acid equivalent (GAE), but its contains a significant amount of catechic tanins and it has been recorded at approximately 31.3 ± 0.01 mg.g^−1^ catechin equivalents (CE). The condensed tannins (non-hydrolyzable tannnins) derive from the condensation of flavan-3-ol monomers such as catechin and/or epicatechin. The identification of these phenolic polymers is carried out by a chemical reaction which is added a few drops of ferric chloride solution (FeCl^3+^) to the crude phenol extract. We note that the seed extracts from date palm show a positive response. The presence of catechin tannins is revealed by a black to intense greenish-black precipitate. However, this tests conducted to demonstrate the presence of hydrolysable tannins showed a negative reaction, leading to the conclusion, date palm seeds of Bent Kbala cultivars do not contain hydrolysable tannins. Few studies have been done on the composition of catechin tannins in the date palm seeds, a major by-product of the date.

Furthermore, cytohistological techniques have already revealed them in seeds teguments of some other date cultivars such as Deglet Nour and Takerbucht [24]. We have shown that anthocyanidins from date seeds inhibit the proliferation of some endophyte fungi from five special fungi from *Fusarium oxysporum* spice [15], which causes significant economic damage to cultivated plants and the environment.

Tannins are generally used in the case of venous insufficiency, hemorrhoidal symptoms, oral hygiene, as antihemorrhagic, anti-dysenteric, cardiac erethic disorders and sleep disorders. Tannins are widely used in the skin tanning industry in industrial applications, but we must note their great importance in food industries such as cider and fruit juice.

### Flavonic aglycones and phenolic acids identified by TLC

In our previous work [15], the determination of the quantitative content of flavonic aglycones was carried out. The TLC of organic extracts (ether phase) provided a good separation of the molecules and an acceptable visibility of the spots under UV (wood light). By calculating the frontal ratios (FR) and taking into account the fluorescence of each revealed compound, we detected the presence of five phenolic compounds (Table 1). The substances are numbered in ascending order of their revealed frontal ratios (FR) at 365 nm (Band I) and 254 nm (Band II). All these results show that the organic extract shows brownish yellow spots observed below 245 nm after spraying with NH^3+^ corresponding to phenolic acids; the UV fluorescence of which is dark blue below 365 nm. Also, aglycone flavonol emit a yellow fluorescence of less than 365 nm.

### Phenolic compounds profiled by HPLC-DAD

The biochemical investigation of natural extract from date seeds by chromatography by diode cluster discovery (HPLC-DAD) at distinctive wave-lengths shows the proximity of seventeen (17) phenolic compounds, of which thirteen (13) are identified at 200 nm, ten (10) at 280 nm, nine (9) at 230 and/or 355 nm, and five (5) at 254 nm. The following families and classes belong to these phenolic compounds (Table 2):

The chromatographic analysis by HPLC-DAD in organic seed extracts is revealed: Quercetol and Isorhamnetin, these molecules detected and identified in seed samples are already found in palm leaves of date cultivars. In the seeds organic extract, a flavone was found, it is Apigenin. The date palm leaves, have already shown this flavone but in their C- glycosylated form, Vitexin [25].

For the first time in the date palm, a new phenolic compound is highlighted, we note that this compound is detected at all wavelengths used. It corresponds to O-glycosylated flavanone, commonly referred to as Prunin or Naringenin 7-O-glucoside. A Quercetol 3-O-rutinoside is a glycosylated flavonol compound, is showed for the first time in the date palm seed.

HPLC-DAD analysis has revealed phenolic acids whose basic skeleton is C_6_–C_1_ or C_6_–C_3_, these compounds are: Six phenolic acids of the benzoic series with a C_6_–C_1_ basic skeleton, derived from benzoic acid, were identified in the analyzed seed samples, namely hydroxybenzoic acids:

Five phenolic acids derived from coumaric acid (C_6_–C_3_) was identified such as trans- cinnamic, hydroxycinnamic, caffeic, dihydroxycinnamic (isomers of caffeic acid) and ferulic acid. A hydroxyquinone was detected at 200 nm, it is an organic compound derived from benzoquinone (single phenol). In many food and medicinal plants, phenolic acids have antioxidant and anti-inflammatory effects and are considered non-toxic during ingestion [26]. Many of these phenolic acids were also found in Algerian seeds Cultivars Deglet Nour and Takerbucht [24]. The edible part of the date of the pulp shows the presence of certain phenolic acids, such as protocatechuic, vanilla, syringic, ferulic, gallic, hydroxybenzoic, caffeic and coumaric acid [27]. Although some Algerian date cultivars some cinnamic acid derivatives have been identified such as ferulic, coumaric, sinapic, 5-o-caffeoyl-shikimic acids and some cinnamic acid derivatives [28]. In many plant species, flavonoids are found in leaves, flowers, pollen and fruits.

Their concentration increases with sun exposure, thus providing protection against photo-and thermo-degradation [29]. For a long time, flavonoids have been revealed in the in the leaves of date palm *Phoenix dactylifera* [25, 30], we remember that some of these compounds have been found for the first time in the date palm seeds: Naringinin and Rutin. Flavonoids generally have the ability to inhibit the oxidation of LDL cholesterol (low-density lipoproteins) and have had significant cardioprotective effects [31].

Quercetin, for example, is a powerful antioxidant, better than ascorbic acid (vitamin C), as it inhibits linoleic acid oxidation [32]. Quercetin also carries out biological activities in the prevention and treatment of hypertension and endothelial dysfunction in rats with spontaneous hypertension [33], as well as in rats with hypertension caused by chronic inhibition of nitric oxide synthase [34], or in rats with renewable hypertension [35]. The antioxidant and chelating ability of flavonoids is attributed to most of the biological activities. Epidemiological studies have shown, among other things, that flavonolrich foods reduce the risk of coronary heart disease mortality [36]. Several studies have shown that a flavonoid-rich diet can have beneficial health effects. Rutin, also known as rutoside, quercetin-3-O- rutinoside or sophorin, is a glycoside that combines Quercetin (flavonol) and rutinose (disaccharide).

Rutin is also an antioxidant that acts on the inhibition of LDL oxidation compared to Quercetin, Apigenin, Kaempferol and Luteolin [37]. *In vitro*, Rutin acts as an angiogenesis inhibitor and inhibits the growth of very high concentrations of vascular endothelial tissues [38]. Rutin inhibits the aggregation of platelets and reduces capillary permeability, thinning the blood and improving circulation [39]. In most plants, isorhamnetin is a neuroprotective compound used to treat brain disorders, neurosensory syndrome, peripheral blood flow disorders and brain failure [40]. In phenylpropanoids derived from cinnamic acid, numerous cis forms have been found, such as cis-para-cou-maric, cis-ferulic and cis-caffeic acids in plants. In mammals, such as acids, hydroxycinnamic acid (para-4-hydroxycinnamic acid) has antioxidant properties and may reduce the risk of abdominal cancer [41].

### Anti-radical activity (scavenger effect) of DPPH

After the addition of DPPH solution, we noticed a dissipation of the initial purple color, in the organic extract seeds to yellow color. Also a positive response to the DPPH test was observed with anti-oxidants molecules (ferulic, gallic, citric, L-ascorbic acids and alpha- tocopherol), there is a significant variation in this antioxidant activity (p > 0.05). However, there is no antioxidant activity was registered in macerate seed (BK). The antioxidant results are expressed as a percentage (Table 3).

Date palm seed extract showed the highest inhibition rate (89.89 ± 0.02%) and alpha- tocopherol (84.4 ± 0.04%) a powerful antioxidant vitamin. The anti-radical power of gallic acids (70.5 ± 0,156%), L-ascorbic acids (61.5 ± 0.04%) and citric acids (62.6 ± 0.15%) is less than those observed previously in seeds extract and vitamin E. Ferulic acid, a reference phenol acid with the lowest anti-radical power (48.73 ± 0.173%). From these data (Table 3), each extract was determined graphically at a concentration of 50% (IC_50_) of the radical DPPH.

This concentration is 0.031 μg mL^−1^ for organic seed extract, so the organic extract BK requires the lowest concentration complex with 50 percent of the free radical DPPH (IC_50_ = 0.031 μg mL^−1^), the anti-oxidant activity is in fact closely related to the extract concentrations because the lowest IC_50_ shows the most important antioxidant activity compared to other standard antioxidants.

The synergistic interactions between antioxidants in the mixture depend not only on the concentration of antioxidants, but also on the structure and nature of the biological activity involved [42]. Polyphenols appear to be effective suppliers of hydrogen to the DPPH radical due to their chemical structure [43].

They act primarily as primary antioxidants to stabilize the peroxide radicals, but they can also deactivate reactive oxygen species such as super oxide ions (O_2_^●−^), hydroxyl radicals OH^●^ or singlet oxygen [44]. In fact, in particular, phenolic compounds and flavonoids are recognized as potentially antioxidant, the scavenger effect of flavonoids (FLOH) is attributed to their low potential redox, which allows the transfer of hydrogen atoms through hydroxyl groups and stable radical molecules (RH) to reduce free radicals (R^●^) [45]. Middleton and co-workers [46] showed that free radical scavenging depend on of several structural factors, such as the most effective flavonoids containing 3′,4′ dihydroxyl groups on ring B and/or a 3-OH group on cycle C.

The presence of free OH in position 3 on the C ring is favorable for antioxidant activity and, for the same molecule, its glycosylation leads to a decrease in activity, which is more pronounced when sugar is a diholoside [44, 46].

## Conclusion

This work initiates the investigation of active compounds from date palm seeds (sub-product of *Phoenix dactylifera*) with antioxidant interest. The extraction of organic fractions and biochemical characterization using spectrophotometers UV-Visible, TLC and HPLC-DAD allowed the identification of a very diverse range of phenolic compounds, such as flavonoids, three flavonols (quercetin, isorhamnetin and kaempferol), four flavones (luteolin, chrysoeriol, tricin and apigenin), However, a flavanone (prunine or naringenin 7-O-glucoside) and an O- glycoside (rutin) are identified for the first time in date palms seeds. Phenolic acids have also been identified, such as *p*-hydroxybenzoic, salicylic, resorcylic, *m*-anisic, syringic and 3,4,5- trimethoxybenzoic (C_6_–C_1_) and five acids listed below, caffeic, dihydroxycinnamic and ferulic (C_6_–C_3_), and a single phenol: Benzoquinone (hydroxyquinone).

The organic seed extract from Bent Kbala cultivar has the highest antioxidant activity (IC_50_ = 0.031 μg.mL^−1^) compared to other antioxidants in relation to the antioxidant activity. In the future, it would also be interesting to consider the valuation of the date product (pulp) and sub-product (seed), which enables different antioxidant molecules to be produced. This would enable concentrated food (powder or granules) to be produced in accordance with scientific standards.

## Figures and Tables

**Table 1:** Various flavonoids revealed and detected in organic extracts of date seeds using Thin-layer chromatography (TLC).

Compound	Frontal ratio (FR)
λ = 245 nm	
1	0.37 ± 0.02
2	0.55 ± 0.05
3	0.84 ± 0.007
4	0.92 ± 0.021
λ = 365 nm	
1	0.27 ± 0.02

**Table 2:** Main phenolic compounds detected at different wavelengths and identified by HPLC-DAD in the organic extract of date palm seeds.

Compound N°	Retention time (RT)	IUPAC nomenclature	Wavelength λ_max_ (nm)
1	3.736	Hydroxyquinone	200
2	6.468	Acid para-hydroxybenzoic	200, 280
3	6.657	Resorcylic acid	200, 230, 254
4	7.070	Caffeic acid	254
5	7.251	Dihydroxycinnamic acid	200, 230, 254, 280
6	8.647	Rutin	200, 230, 280
7	8.781	Hydroxycinnamic acid	200, 230, 280, 355
8	9.273	Ferulic acid	200, 230, 280, 355
9	9.627	Syringic acid	200
10	9.897	Salicylic acid	355
11	10.377	Naringenin 7- O - glucoside	200, 230, 254, 280, 355
12	10.899	Acid 3,4,5 trimethoxybenzoic	200, 230
13	11.828	m- anisic acid	280, 355
14	12.873	Quercetin	200, 230, 280, 355
15	13.840	Trans-cinnamic acid	254, 280, 355
16	14.458	Apigenin	200, 230, 280, 355
17	15.080	Isorhamnetin	200, 355

**Table 3:** Results of the free radical inhibition test and extracts studied.

Compounds	Antioxidant activity (%)	IC_50_ (μg.mL-1)
Seeds	84.97 ± 0.024	0.031 ± 0.033
*α*-Tocopherol	84.40 ± 0.040	1.50 ± 0.43
Gallic acid	70.50 ± 0.156	3.21 ± 0.71
Citric acid	62,60 ± 0,150	1.79 ± 0.12
Acid L-ascorbic	61.53 ± 0,040	2.50 ± 0.53
Ferulic acid	48.73 ± 0.173	4.96 ± 0.17
Macerat	-	-
